# Identification of stable, high copy number, medium-sized RNA degradation intermediates that accumulate in plants under non-stress conditions

**DOI:** 10.1007/s11103-013-0079-3

**Published:** 2013-05-25

**Authors:** Martyna Nowacka, Pawel M. Strozycki, Paulina Jackowiak, Anna Hojka-Osinska, Maciej Szymanski, Marek Figlerowicz

**Affiliations:** 1 Institute of Bioorganic Chemistry, Polish Academy of Sciences, Noskowskiego 12/14, 61-704 Poznan, Poland; 2 Computational Genomics Laboratory, Institute of Molecular Biology and Biotechnology, Adam Mickiewicz University, Umultowska 89, 61-614 Poznan, Poland; 3 Institute of Computing Science, Poznan University of Technology, Piotrowo 3A, 60-965 Poznan, Poland; 4 Present Address: Laboratory of Bioinformatics and Protein Engineering, International Institute of Molecular and Cell Biology in Warsaw, Trojdena 4, 02-109 Warsaw, Poland

**Keywords:** RNA degradome, 2D-PAGE, Small regulatory RNA, Translation inhibition

## Abstract

It is becoming increasingly evident that the RNA degradome is a crucial component of the total cellular RNA pool. Here, we present an analysis of the medium-sized RNAs (midi RNAs) that form in *Arabidopsis thaliana*. Our analyses revealed that the midi RNA fraction contained mostly 20–70-nt-long fragments derived from various RNA species, including tRNA, rRNA, mRNA and snRNA. The majority of these fragments could be classified as stable RNA degradation intermediates (RNA degradants). Using two dimensional polyacrylamide gel electrophoresis, we demonstrated that high copy number RNA (hcn RNA) degradants appear in plant cells not only during stress, as it was earlier suggested. They are continuously produced also under physiological conditions. The data collected indicated that the accumulation pattern of the hcn RNA degradants is organ-specific and can be affected by various endogenous and exogenous factors. In addition, we demonstrated that selected degradants efficiently inhibit translation in vitro. Thus, the results of our studies suggest that hcn RNA degradants are likely to be involved in the regulation of gene expression in plants.

**Electronic supplementary material:**

The online version of this article (doi:10.1007/s11103-013-0079-3) contains supplementary material, which is available to authorized users.

## Introduction

The small regulatory RNAs identified thus far in plants can be divided into three major classes: small interfering RNAs (siRNAs), microRNAs (miRNAs) and *trans*-acting small interfering RNAs (ta-siRNAs) (Bonnet et al. 2006). These small RNAs control essential cellular processes by regulating development and homeostasis and defending against retrotransposons and viral infections (Johnson and Sundaresan 2007; Bonnet et al. 2006). It has become increasingly evident that the range of known, potentially functional, non-coding RNAs is likely to expand and includes RNA degradation intermediates that have not been considered so far (Jackowiak et al. 2011). RNA degradation is a highly efficient process that is associated with RNA maturation, quality control and turnover. This high efficacy ensures that gene expression pathways are not affected by accidentally formed RNA molecules (Houseley and Tollervey 2009). However, it has been demonstrated that the degradation of several types of mature RNAs (including tRNAs, rRNAs and snoRNAs) can yield stable intermediates (Thompson et al. 2008; Taft et al. 2009; Zhang et al. 2009) that together constitute the RNA degradome (Jackowiak et al. 2011). The production of these relatively short (usually 20–70-nt) molecules is postulated to be mostly associated with developmental processes and the response to stress conditions (Emara et al. 2010; Thompson et al. 2008; Haiser et al. 2008). Because stable degradation intermediates have been reported across kingdoms of life, the mechanisms of their generation are likely to be ancient and evolutionarily conserved. Although data concerning the functionality of degradation intermediates are limited, these molecules have been postulated to act as gene expression regulators in both plants and animals (Zhang et al. 2009; Lee et al. 2009; Haussecker et al. 2010; Li and Zhou 2009; Elbarbary et al. 2009; Yamasaki et al. 2009). In addition, degradants in the phloem sap of pumpkin (*Cucurbita maxima*), which originated from tRNAs, rRNAs and/or snoRNAs, were proposed to undergo long-distance transport, participate in plant-signaling mechanisms and inhibit in vitro translation (Zhang et al. 2009). Other possible functions of RNA degradants have recently been discussed in several review papers (Aalto and Pasquinelli 2012; Hurto 2011; Sobala and Hutvagner 2011; Tuck and Tollervey 2011; Thompson and Parker 2009; Pederson 2010). In addition, it has also been thought that products of RNA degradation can interfere with regulatory pathways for example by recruiting and sequestering important proteins or regulatory complexes (Buhler et al. 2008). As a result, these RNAs could compete with other regulatory molecules, likely miRNAs or siRNAs. Additionally, growing evidence that short RNAs in plants operate in cascades (Chen et al. 2007; MacLean et al. 2010) suggests that RNA degradants may also function as transitory molecules involved in the production or activation of other regulatory factors, in transmitting gene-silencing signals to other genes or in guiding some molecules to their final regulatory destination.

Lately, we have demonstrated that plant cells contain a large group of 10–90-nt-long RNAs that repeatedly form and accumulate to considerable levels (Nowacka et al. 2012). Importantly, beside si/miRNAs and tRNAs, this group includes many molecules of unknown origin and function. Based on their lengths, these poorly studied RNAs can be divided into two subgroups. The first group contains molecules that are shorter than si/miRNAs, and the second group comprises molecules that are longer than si/miRNAs but shorter than tRNAs (i.e., medium-sized RNA; abbreviated midi RNA). Lately, Hsieh et al. (2009, 2010) have identified RNAs that are shorter than si/miRNAs and form in *Arabidopsis thaliana* upon phosphate starvation. These 19-nt-long molecules corresponded to the 5′ ends of tRNAs and constituted approximately one-third of the small RNA pool in phosphate-deficient roots. Interestingly, these RNAs accumulated to much lower levels in the shoots. Based on this observation, the authors postulated that these molecules were products of a specific cleavage rather than of random RNA decay. In addition, Hsieh et al. (2010) hypothesized that an unequal distribution of the identified tRNA fragments can result from long-distance transport of these molecules, which serve as signals of the metabolic state of the cell/tissue. The only midi RNAs that have been reported in Arabidopsis thus far are tRNA halves that resulted from anticodon loop cleavage (Hsieh et al. 2010; Thompson et al. 2008). These molecules are 30–55-nt long and their accumulation seems to be connected with a reaction to imminent stress, such as nutrient deficiency or oxidative stress. The data collected indicated that tRNA halves in plants act not only as signal transducers but also as translation inhibitors (Thompson et al. 2008; Zhang et al. 2009; Hsieh et al. 2009). Earlier, we showed that two-dimensional polyacrylamide gel electrophoresis (2D-PAGE) enables the analysis of RNAs that range in length from 10 to 90 nt (Nowacka et al. 2012). Accordingly, we have used this technique to determine the pattern of accumulation of high copy number midi RNAs (hcn-midi RNAs) in plant and human cells. We have demonstrated that under constant conditions, this pattern is stable and organ- or cell-specific. In addition, our data suggested that some hcn-midi RNAs were stable RNA degradation intermediates, i.e., fragments of tRNA, rRNA, mRNA and snRNA (Nowacka et al. 2012).

In this study, we attempted to better characterize the hcn-midi RNAs that accumulate in the model dicotyledonous plant *Arabidopsis thaliana*. We identified 59 RNA degradants that were 17–118-nt long and accumulated in plant leaves to levels that were similar to those of miRNAs. We determined the putative precursors of RNA degradants and showed that some of the degradants can inhibit translation in vitro. In addition, we performed comparative analyses of hcn-midi RNAs that accumulated in different Arabidopsis organs, in Arabidopsis leaves after exposure to abiotic stress and in leaves of Arabidopsis mutants with impaired pathways of small RNA biogenesis. The results obtained indicate that specific accumulation profiles of hcn-midi RNAs could be ascribed to individual plant organs and physiological stages.

## Materials and methods

### Plant growth

Wild type and mutant *Arabidopsis thaliana* (ecotype Columbia-0) plants were grown under short-day, standard conditions as described previously (Nowacka et al. 2012). Leaf and root samples were collected from 5-week-old plants, and flower samples were collected from 7-week-old plants. The *dcl1*-*5* mutant, which exhibited a slow-growth phenotype, was an exception. In this case, the plants were grown for 7–8 weeks before the leaves were collected. Osmotic stress was induced by watering wild type plants with a 150 mM NaCl solution. Leaves were collected from plants after a 6 h exposure to salinity, and all samples were immediately frozen in liquid nitrogen and stored at −80 °C. The following lines of Arabidopsis were used in our experiments: sus1-5 (allele *dcl1*-*5*, locus At1g01040), SALK_018392 (allele *dcl2*-*1*, locus At3g03300), SALK_005512 (allele *dcl3*-*1*, locus At1g43920) and GK-160G05 (allele *dcl4*-*2*, locus At5g20320) (Lamesch et al. 2010).

### Two-dimensional electrophoresis of small RNAs

RNA samples, which were enriched in molecules that were shorter than 200 nt, were isolated from Arabidopsis (leaves, roots or flowers) using the *mir*Vana miRNA Isolation Kit (Ambion). Subsequently, 1 μg of RNA was labeled with γ^32^P ATP using T4 PNK kinase (Fermentas) and subjected to 2D-PAGE.

2D-PAGE was performed under semi-denaturing conditions (4 M urea) as previously described (Nowacka et al. 2012), and 10 % and 20 % polyacrylamide gels were used for first (1D) and second (2D) dimension electrophoresis, respectively. After 1D-separation, an approximately 25 × 2.5-cm gel slice, which contained RNA molecules that migrated slower than an 18-nt RNA marker (si/miRNA size range) and faster than the main band corresponding to tRNA, was excised, placed between new electrophoretic plates and embedded in a freshly prepared, 10 % polyacrylamide gel. The remaining space between the electrophoretic plates was then filled with 20 % polyacrylamide gel, and the 2D-separation was performed. The gel was exposed to a phosphor screen and scanned with a Fuji FLA-5100 imaging system. The intensities of spots visualized in 2D-gel images were quantified with Multi Gauge software. All experiments were performed at least in triplicate.

In order to compare the accumulation of hcn-midi RNA degradants in two samples, the images obtained for these samples were superimposed and matching spots were identified. Next, the individual spot intensities quantified with Multi Gauge software (our raw data) were normalized. To this end, for each pair of images to be compared three spots that displayed invariable intensity in relation to the background were selected (three corresponding spots for both images) and for each image the mean intensity of these three spots (mean count) was calculated. Then, for every spot its relative intensity (relative spot count) was determined by dividing initially quantified intensity by mean count. Finally, the changes in individual degradants’ accumulation were determined by comparison of the relative spot counts of matching spots. The accumulation was considered significantly different when twofold change of relative spot count was observed.

### Construction of cDNA libraries

Two cDNA libraries of RNAs from Arabidopsis leaves were prepared based on the protocols that were elaborated by Lau et al. (2001), Chappell et al. (2006), Lee and Ambros (2001) and as previously described (Nowacka et al. 2012).

The 2D-library was constructed using RNAs that were extracted from individual spots that were visualized on the 2D-gels. For the proper selection of RNA spots, 9 experiments of 2D-separation were performed, each of which involved harvesting Arabidopsis rosette leaves from 3 independent plant cultivations. Subsequently, 3 independent RNA extractions followed by RNA separations in 2D-gels were performed from each plant harvest. Finally, after analyzing the obtained images, gel fragments corresponding to the 70 most intensive spots that were present on all images were chosen and excised. To optimize cloning, fragments that were excised from the gel were divided into three fractions, and each fraction contained pooled gel fragments of similar spot intensity. RNA was eluted from the gel fragments, and equivalent (radioactivity in cpm) portions of each RNA fraction were utilized for cloning.

The second cDNA library (i.e., the 1D-library) was prepared using the RNA that was eluted from 1D-gel slices (parallel slices were used for 2D-separation in 2D-library construction, as described above). The resulting RNA fraction, which was subsequently used for cloning, contained molecules that migrated slower than 18-nt RNA size marker but faster than the main band corresponding to tRNA. Accordingly, most of tRNA molecules were not included in this library.

For both libraries, RNAs that were isolated from the gels were ligated with a 3′ End Donor oligonucleotide, which contained the BanI restriction site, a blocked 3′-hydroxyl terminus and a pre-activated 5′ terminus and was commercially available from IDT, as previously described (Nowacka et al. 2012). The ligation products were separated in a 10 % denaturing polyacrylamide gel, eluted, precipitated with ethanol, and subjected to reverse transcription. The reverse transcription reaction was performed using Superscript II reverse transcriptase (Invitrogen) and the designed primers (each containing the BanI restriction site) (Nowacka et al. 2012). Due to the terminal transferase activity of this enzyme, the second strand of the cDNA was synthesized in the same tube without prior ligation of the 5′ adapter oligonucleotide. The final RT-PCR products were purified by phenol–chloroform extraction, digested with BanI and concatemerized using T4 DNA ligase (Promega). The gel-purified concatemers (in low-melting-point agarose) were ligated into the TOPO-TA cloning vector and introduced into TOP10 electrocompetent cells. Plasmids that contained inserts were isolated from the individual colonies and sequenced using the ABI PRISM 310 Genetic Analyzer.

The sequences of the molecules from both cDNA libraries were mapped onto the reference Arabidopsis genome using BLAST (Altschul et al. 1990). The genomic sequences and four datasets for gene sequences (cDNA, intron, exon and intergenic sequences) were obtained from the TAIR database (Garcia-Hernandez et al. 2002) (www.arabidopsis.org). ORFs, pseudogenes and intergenic sequences were generated using the TAIR9 release of the Arabidopsis genome annotation, and the sequences for which there were no matches in the TAIR9-annotated portions of the genome were used to search the non-redundant set of Arabidopsis sequences that was derived from GenBank.

### *In vitro* translation inhibition by tRNA degradants

Wheat Germ Extract System (Promega) was used for in vitro translation of Luciferase Control RNA (Promega) that encodes functional firefly luciferase. Translation reactions were prepared according to the manufacturer’s recommendations with some modifications. All reactions were carried out in 10 μl final volume with 50 % Wheat Germ Extract, 80 μM Amino Acid Mixture, 8 units of ribonuclease inhibitor RNaseOUT (Invitrogen) and 2 μg Luciferase Control RNA. 10 pmol of chemically synthesized, 5′ monophosphorylated RNA oligonucleotide (IBA) corresponding to the degradant was added to a translation reaction and incubated for 1.5 h at 30 °C. A control reaction with no short RNA added was performed in parallel. After incubation, luciferase activity was quantitated (three independent measurements) with Victor X4 Multilabel Plate Reader (PerkinElmer) and Luciferase Assay System (Promega). For a single measurement 2.5 μl of a translation reaction mixture were used. All reactions were carried out in triplicates.

## Results

### 2D-PAGE profiling of hcn-midi RNAs from Arabidopsis leaves

**Fig. 1:**
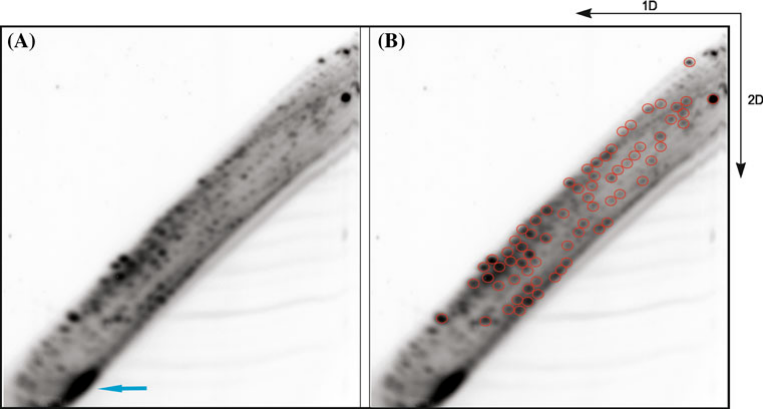
2D-PAGE analysis of the midi RNA fraction that was isolated from Arabidopsis rosette leaves. The directions of electrophoresis in the first and second dimension are indicated by *arrows*. Characteristic pattern of RNA accumulation in Arabidopsis leaves (**a**); *blue arrow* indicates a group of spots that correspond to si/miRNAs. Seventy leaf-specific spots are indicated by *red circles* (**b**). Gel fragments that correspond to the *marked spots* were used for 2D-library construction

To determine how variable is the profile of hcn-midi RNAs accumulation in fully developed plant tissue, we examined RNA that was isolated from mature, developmentally stable Arabidopsis rosette leaves. Three sets of plants were grown under the same conditions (for details, see “Materials and methods”), and after 5 weeks, rosette leaves were collected and frozen at −80 °C. Three samples of RNA, which were enriched in molecules that were shorter than 200 nt, were isolated from each harvest. As a result, we obtained 9 RNA samples that were then 5′-end labeled with ^32^P and subjected to 2D-PAGE analysis. After 1D-separation, the fraction of RNA that migrated slower than the 18-nt-long radiolabeled RNA marker but faster than the majority of tRNAs was subjected to 2D-separation. Upon autoradiography, hcn-midi RNAs and si/miRNAs were visualized (Fig. 1a). The latter localized to the bottom of the 2D-gel and formed a cluster of unseparated spots (Fig. 1a). Subsequently, the amounts of individual midi RNAs were quantified using Multi Gauge software, and comparative analysis of all nine autoradiograms showed that the results were highly reproducible. Consequently, we selected 70 individual spots that were characteristic of all autoradiograms (Fig. 1b). Each of these spots was present in all autoradiograms and displayed a similarly high intensity. Together, all of the selected spots represented a characteristic pattern of midi RNA accumulation in Arabidopsis leaves. We also found that the total intensity of the 70 selected spots was higher than that of the cluster of unseparated spots that corresponded to si/miRNAs. Based on this observation, we assumed that all hcn-midi RNA molecules that were characteristic of Arabidopsis leaves accumulated in cells to similar or higher levels than si/miRNAs.

### Identification of hcn-midi RNAs from Arabidopsis leaves

To identify Arabidopsis rosette leaf-specific hcn-midi RNAs, 70 gel fragments corresponding to spots that represented the characteristic pattern of midi RNA accumulation in Arabidopsis leaves were excised from the 2D-gels (Fig. 1b), RNA was extracted from those fragments and pooled. In parallel, an entire fraction of RNA that migrated slower than the 18-nt-long RNA marker but faster than the majority of tRNAs was extracted from 1D-gel slices. Both RNA pools were used to generate cDNA libraries (for details, see “Materials and methods”). The first library was named the “2D-library” because its elements were selected based on the 2D-gels. The second library was named the “1D-library” because it contained RNA that was extracted from 1D-gels (i.e., the fraction that after separation in a 1D-gel was usually subjected to electrophoresis in a 2D-gel). Thus, the 2D-library represented a subset of the 1D-library. The obtained cDNAs were concatemerized, cloned and sequenced. To prepare the libraries, 3′- but not 5′-adapter ligation was performed (see “Materials and methods”). Therefore, all RNA molecules that possessed 3′-hydroxyl groups and non-phosphorylated or phosphorylated 5′-ends were cloned.

#### 2D-library

**Fig. 2:**
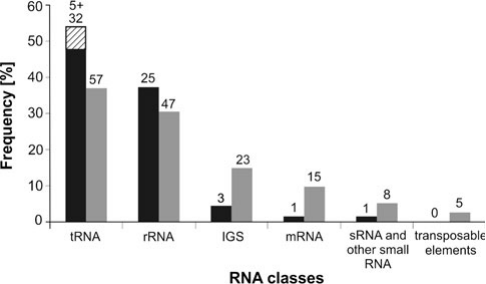
Composition of 2D- (*black*) and 1D-libraries (*gray*). *Cross hatched square* represents full-sized tRNAs

Five hundred concatemers, each comprising 2–3 individual molecules, were sequenced, and as a result, we identified 67 unique molecules. In several cases, the sizes of these molecules varied by a few nucleotides at the 3′- or 5′-end. All of these size variants were grouped together, and only the longest molecule from each group was considered for further analyses. The sizes of the identified molecules ranged from 17 to 118 nt. Comparison of the obtained sequences with the Arabidopsis genome database revealed that the majority of the cloned molecules (i.e., at least 59 hcn-midi RNAs) represented fragments of larger RNA species. Accordingly, these molecules could be classified as intermediates of RNA degradation that accumulated in plant cells (see Online Table 1). Three molecules were classified as unknown transcripts (or fragments of those transcripts) from intergenic regions and 5 were identified as intact tRNA molecules (tRNA^Glu^, tRNA^Gln^, tRNA^Arg^ and 2 tRNA^Asp^). Of 59 putative degradants, 32 molecules were derived from tRNA (47.8 %) and 25 were derived from rRNA (37.3 %) (Fig. 2, Online Figs. 1 and 2). Only 1 mRNA and 1 snRNA fragment were identified. Exclusively for RNAs that were derived from tRNA, several single nucleotide mismatches to the Arabidopsis genomic sequence were detected during the BLAST search. The comparison of the mismatch locations with the pattern of Arabidopsis tRNA modifications revealed that single nucleotide mutations occurred at positions that were normally occupied by a modified adenosine (m1a). Accordingly, one can assume that these mutations were introduced during reverse transcription.

Further analysis revealed that tRNA degradants were derived from only 12 different types of nuclear tRNAs and 10 types of organellar tRNAs (including 2 tRNAs which could hypothetically originate from nuclear as well as organellar genes). We also analyzed how particular tRNA isoacceptors contributed to the degradome. We found fragments of 4 tRNA^Leu^ and 4 tRNA^Gly^ isoacceptors and fragments of 3 tRNA^Glu^ and 3 tRNA^Arg^ isoacceptors (Table 1 and Online Fig. 1). Interestingly, the majority of the identified tRNA fragments (75 %) corresponded to the 3′ portion of the full-length molecule. Among these fragments, we found 3′ halves and 3′ quarters of tRNAs, degradants that were comprised of three-quarters of the full-sized molecule and nearly complete, 5′-trimmed tRNAs (Table 2). Almost all of these degradants (83.3 %) contained an intact CCA sequence (or trimmed –CC or –C nucleotide variants) at their 3′ ends. Moreover, none of the tRNA degradants contained 5′ leader, 3′ trailer or intronic sequences. Degradants that corresponded to the 5′ portion of tRNAs were less frequent (25 %) and included 5′ halves that were predominantly trimmed at their 5′ ends (Table 2). In two cases, fragments that represented both halves of the same tRNA (tRNA^Gly^ and tRNA^Leu^) were identified in the 2D-library.

**Table 1:** Assignment of the RNA fragments identified in 1D- and 2D-libraries to tRNA isoacceptors in the *Arabidopsis thaliana* genome

Amino acid	No. of tRNA isoacceptors in Arabidopsis genome	Putative precursors of tRNA degradants identified in 1D-library			Putative precursors of tRNA degradants identified in 2D-library		
		No. of identified degradants	Number of precursors	Anticodons and genes coding for precursors	No. of identified degradants	Number of precursors	Anticodons and gene symbols of representative sequences
Ala	8	5	6	AGC: AT1G06610, AT4G11355 CGC: AT1G64420, AT2G22580, AT3G61755 TGC: ATCG00940	1	2	AGC: AT1G06610, AT4G26675
Arg	23	6	10	CCG: AT1G16450 CCT: AT1G49690, AT4G34415, AT5G10525 TCG: AT1G79980 TCT: AT2G45020, AT3G10035, AT4G34035, AT5G03775 ACG: ATCG00980	3	3	ACG: AT1G13010, AT2G22280 CCG: AT1G16450
Asn	8	2	2	GTT: AT2G07764, ATCG01140	0	0	
Asp	6	4	5	GTC: AT1G03515, AT3G51265, AT2G07743, AT2G33650, ATCG00230	1	1	GTC: ATCG00230
Cys	12	4	4	GCA: AT1G53410, AT1G63510, AT2G39600, ATCG00200	2	2	GCA: AT1G53410, AT3G52345
Gln	8	0	0		1	1	TTG: ATCG00060
Glu	12	3	4	CTC: AT1G29210, AT2G38030 TTC: AT1G75970, ATCG00250	3	3	CTC: AT1G29210 TTC: AT3G05525, ATCG00250
Gly	12	8	10	CCC: AT2G47740 GCC: AT1G04320, AT1G06860, AT1G60840, AT1G60910, AT1G71700, AT3G06105, AT5G02025, ATCG00310 TCC: AT1G08240	4	9	CCC: AT2G47740 GCC: AT1G04320, AT1G06860, AT1G60840, AT1G60910, AT1G71700, AT3G06105, AT5G02025, ATCG00310
His	4	1	1	GTG: ATCG00010	2	2	GTG: AT1G02600, ATCG00010
Ile	5	2	1	GAT: ATCG00930	2	2	AAT: AT1G06480 GAT: ATCG00930
Leu	17	4	7	AAG: AT1G74570, AT5G60285 TAG: AT1G61910, AT2G36150 CAA: ATCG01260, ATCG00880 CAG: ATCG01030	4	6	TAG: AT1G61910, AT2G36150 TAA: ATCG00400 CAA: ATCG01260, ATCG00880 CAG: ATCG01030
Lys	9	2	4	CTT: AT1G01890 TTT: AT1G79290, AT3G12385, AT4G28362	0	0	
Met	12	1	1	CAT: AT2G07755	0	0	
Phe	3	1	1	GAA: ATCG00410	1	2	GAA: AT1G02480, AT1G68950
Pro	14	2	4	AGG: AT1G28820, AT1G28870 CGG: AT2G21360, AT3G05755	0	0	
Ser	28	1	3	GGA: AT2G07757 TGA: AT1G76330, AT3G07115	1	2	CGA: AT4G32765 GGA: ATCG00370
Thr	11	3	2	AGT: AT1G49280 TGT: AT1G05980	1	1	GGT: ATCG00260
Trp	4	2	3	CCA: AT1G11640, AT1G20820, AT2G07748,	2	4	CCA: AT1G11640, AT1G20820, AT2G07748, ATCG00610
Tyr	33	3	5	GTA: AT1G57170, AT2G07765, AT2G07792, AT3G20365, ATCG00240	2	2	GTA: AT1G57170, AT3G48515
Val	8	3	5	AAC: AT1G17670 TAC: AT1G01870, AT3G50835, AT4G16235 GAC:	0	0

**Table 2:**
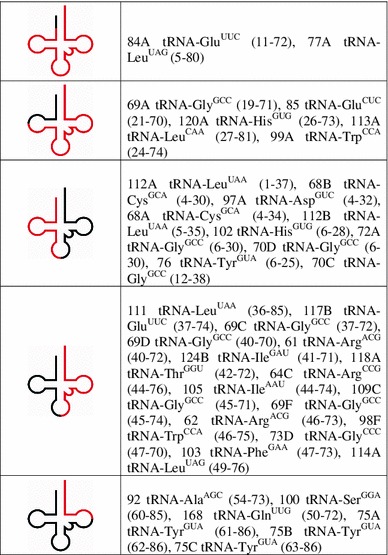
tRNA fragments (in red) identified in 2D-library −75 % of them correspond to the 3′ and 25 % to the 5′ portions of the full-sized molecules

Numbers in brackets indicate positions of the first and the last nucleotide of a given tRNA fragment in its precursor tRNA (positions are counted from 5′ to 3′; CCA motifs are not considered)

The second most abundant class of RNA degradants in the 2D-library (25 molecules) consisted of nuclear and organellar rRNA fragments. The vast majority of rRNA fragments were derived from 25S rRNA (10 molecules) and 18S rRNA (9 molecules). The remaining fragments corresponded to 5S rRNA (3 molecules), 23S rRNA (1 molecule) and 16S rRNA (2 molecules) (Online Fig. 2). One hcn-midi RNA from the 2D-library was identified as a derivative of the U2.3 small nuclear RNA, and only one sequence was identified as a fragment of an mRNA. The latter was a fragment of the 5′ untranslated region (UTR) of the mRNA that encodes a RING/U-box superfamily protein (AT4G39140).

#### 1D-library

Each of the 350 sequenced concatemers from the 1D-library contained 2–7 individual molecules. As a result, 154 unique RNAs were identified and the size variants were grouped as described above for the 2D-library. The length of these molecules ranged from 15 to 47 nt, and as expected, nearly all of the cloned molecules (except for 7 (4.5 %) si/miRNA molecules) were the putative products of RNA degradation (Online Table 2). Once again, most of these fragments were classified as tRNA (37 %; 57 molecules) or rRNA (30.5 %; 47 molecules) derivatives (Fig. 2). Additionally, we identified fragments of unknown transcripts that were encoded in the intergenic regions (14.9 %; 23 molecules), fragments of mRNAs (9.7 %; 15 molecules), and transcripts that originated from transposable elements (2.6 %; 4 molecules) and snRNAs (0.7 %; 1 molecule) (Fig. 2).

#### Comparison of 1D- and 2D-libraries

The 1D-library included high and low copy number midi RNAs, but the 2D-library contained only hcn-midi RNAs. If the same mechanisms are involved in high and low copy number midi RNA formation, the compositions of both libraries should be similar. It means that the libraries should contain the same fractions of degradants that are derived from particular classes of RNA (e.g. tRNA, rRNA and mRNA). To verify this presumption we compared the structures of both libraries. More than half of the RNA degradants from the 2D-library (36 RNAs; including size variants) was also found in the 1D-library. Among these RNAs were fragments of 19 tRNAs, 15 rRNAs and 2 intergenic sequences (IGS). Two types of RNA: fragments of transcripts of transposable elements and si/miRNAs were present only in the 1D-library (Fig. 2). In general, the 2D-library was dominated by fragments of tRNA, rRNA, and contained only minor amounts of other RNAs (together, approximately 15 %). The composition of the 1D-library was more balanced, and a significant portion of the library (approximately 30 %) consisted of derivatives of molecules other than tRNA and rRNA.

### Inhibition of in vitro translation by selected tRNA degradants

**Fig. 3:**
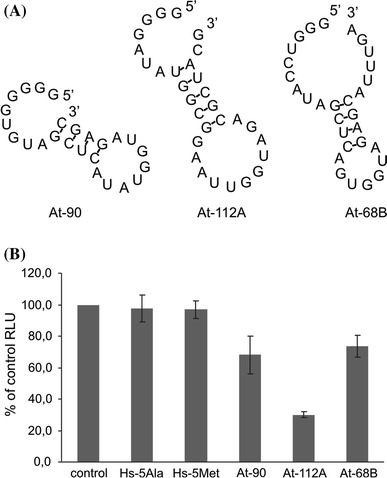
Inhibition of in vitro translation by tRNA degradants. **a** Secondary structures of selected tRNA degradants identified in 2D- (At-112A, At-68B) and 1D- (At-90) libraries, predicted by RNAfold. **b** Uncapped firefly luciferase mRNA was translated in vitro using wheat germ extract in the presence of tRNA degradants from human cells (Hs-5Ala, Hs-5Met) and Arabidopsis leaves (At-90, At-112A and At-68B). Luciferase activity in the absence of added short RNA (control) is assigned as 100 % and luciferase activity in the presence of degradants is relative to control. Means and SD are from three replicates in three independent experiments. *RLU* relative light unit

One of the functions proposed for tRNA fragments is the inhibition of translation. Ivanov et al. (2011) demonstrated that in a rabbit reticulocyte lysate reporter mRNA translation was inhibited by tRNA degradants that: (1) had 5′ oligo-guanine (TOG) motif and (2) were predicted by RNAfold to have a single stranded 5′ region, followed by a stem-loop and a single stranded 3′ region. We identified similar degradants in 2D- (degradants no. 68B and 112A) and 1D- (degradant no. 90) libraries (Fig. 3a). To determine if they are also capable of affecting protein synthesis, we tested their influence on in vitro translation of reporter mRNA in a wheat germ extract. In addition, we used 5′ fragments of human tRNA^Ala^ (Hs-5Ala, 30 nt) and tRNA^Met^ (Hs-5Met, 31 nt). The former fulfilled both structural criteria mentioned above and was shown to be an effective translational inhibitor in rabbit reticulocyte lysate, while the latter lacked a 5′ TOG motif and was unable to block translation in the same system (Ivanov et al. 2011). In our assay, uncapped firefly luciferase mRNA that encodes an active enzyme was translated in vitro in the wheat germ extract in the presence of selected degradants (chemically synthesized oligonucleotides). Subsequently, luciferase activity was quantitated. In case of all three degradants from Arabidopsis a significant drop in luciferase activity was detected as compared to a control reaction with no RNA degradant added (*p* < 0.00005, Fig. 3b), thus indicating translational arrest. This effect was observed at a relatively low concentration of degradants (1 μM). The 5′ fragment of human tRNA^Ala^ (effective inhibitor in a mammalian system) and the 5′ fragment of human tRNA^Met^ (inactive in a mammalian system) did not block translation in the plant in vitro system. Among the Arabidopsis degradants no. 112A (tRNA^Leu^) was the strongest repressor and caused approximately 70 % decrease in translation efficiency. The other two degradants reduced translation by c.a. 32 % (no. 90, tRNA^Ala^) and 26 % (no. 68B, tRNA^Cys^).

### Examples of endogenous and exogenous factors that may affect the accumulation pattern of hcn-midi RNAs

After characterizing the specific accumulation patterns of hcn-midi RNAs in leaves, we attempted to determine the extent to which this pattern was organ-specific. To this end, RNA samples isolated from Arabidopsis roots and flowers were subjected to 2D-PAGE analysis in the same way as the rosette leaf samples. Subsequently, the obtained gel images were compared (Online Fig. 3). In all performed comparative analyses the pattern of the previously selected 70 spots, which corresponded to hcn-midi RNAs from rosette leaves, was applied as a reference.

**Fig. 4:**
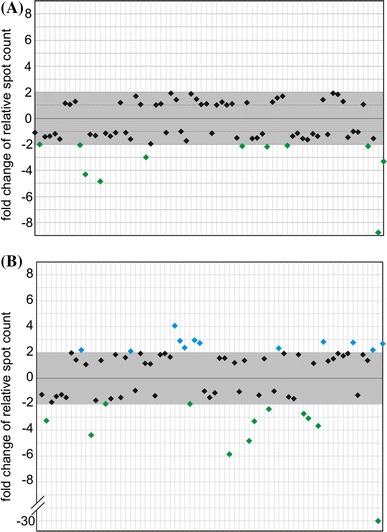
The relative levels of hcn-midi RNA accumulation in Arabidopsis leaves, roots and flowers. Changes in the hcn-midi RNA accumulation levels: **a** roots versus leaves. (b) flowers versus leaves. The range of changes considered to be insignificant (lower than twofold) is marked with a *gray box*. Each *spot* is represented by a separate *diamond symbol* (*spots* 1–70 are arranged consecutively from *left* to *right*). In order to determine the fold changes of hcn-midi RNA accumulation levels, the relative *spot* counts obtained for roots or flowers were divided by corresponding relative spot counts obtained for leaves. *Spots* that displayed significantly higher relative intensity in leaf profile are *colored green* (**a**, **b**). *Spots* that displayed significantly higher relative intensity in flower profile (**b**) are *colored blue*. No spots that exhibited significantly higher relative intensity in root profile were detected (**a**)

Quantitative analysis of leaf and root samples revealed that only 11 degradants displayed a significantly altered level of accumulation. All of them were more prevalent in leaves (Fig. 4a). Comparison of leaf and flower samples demonstrated 24 significantly differentiating degradants—half of them accumulated to higher levels in leaves, whereas the other half in flowers (Fig. 4b). Among the degradants that displayed significant changes in the levels of accumulation in both comparisons, 6 were common—5 of them were more abundant in leaves than in roots or flowers, and 1 had lowest level of accumulation in roots, higher in leaves and highest in flowers. Our results indicated that most degradants are constitutively present and similarly accumulate in different organs (leaves, roots and flowers). However, there exists a number of hcn-midi RNAs which display organ-specific differences in accumulation levels.

**Fig. 5:**
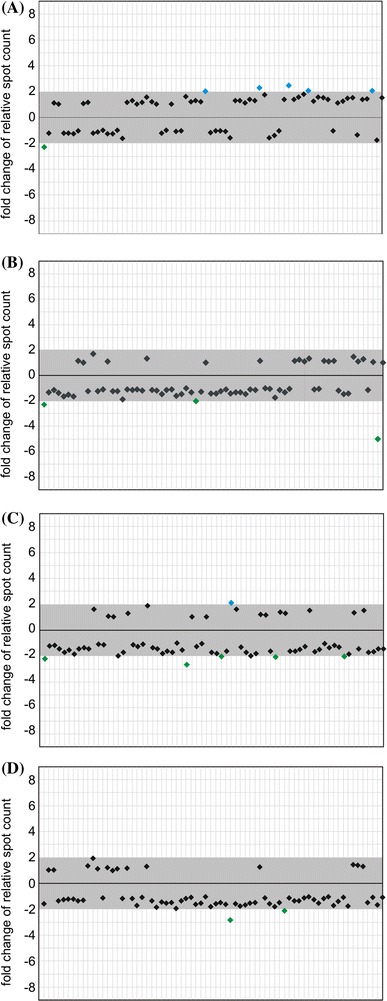
The relative levels of hcn-midi RNA accumulation in wild type and mutant Arabidopsis leaves. Changes in the hcn-midi RNA accumulation levels: *dcl1*-*5* versus wild type (**a**); *dcl2*-*1* versus wild type (**b**); *dcl3*-*1* versus wild type (**c**); *dcl4*-*2* versus wild type (**d**). The range of changes considered to be insignificant (lower than twofold) is marked with a *gray box*. Each spot is represented by a separate *diamond symbol* (spots 1 to 70 are arranged consecutively from *left* to *right*). In order to determine the fold changes of hcn-midi RNA accumulation levels, the relative spot counts obtained for mutant leaves were divided by corresponding relative spot counts obtained for wild type leaves. Spots that displayed significantly higher relative intensity in wild type leaf profile are *colored green* (**a**–**d**). Spots that displayed significantly higher relative intensity in *dcl1*-*5* and *dcl3*-*1* are *colored blue* (**a**, **c**, respectively). No spots that exhibited significantly higher relative intensity in *dcl2*-*1* and *dcl4*-*2* profiles were detected (**b**, **d**, respectively)

We also attempted to determine if the RNAi machinery is involved in the generation of hcn-midi RNAs. Therefore, we examined whether the biogenesis of these molecules is dependent on the presence of Dicer-like (DCL) ribonucleases. We performed 2D-PAGE analyses of RNA samples that were isolated from rosette leaves of four Arabidopsis mutants, each lacking one of the DCLs. Earlier, it was shown that these mutants display an aberrant accumulation of miRNAs, siRNAs and tasiRNAs because of defects in small RNA biogenesis pathways (Chapman and Carrington 2007). In addition, mutants devoid of DCL1 or DCL4 exhibit complex developmental defects (Xie et al. 2005; Schauer et al. 2002). Comparison of the hcn-midi RNAs of DCL mutants (*dcl1*-*5*, *dcl2*-*1*, *dcl3*-*1* and *dcl4*-*2*) and wild type plants (Fig. 5, Online Fig. 4) revealed the following numbers of significantly differentiating degradants: 6 for *dcl1*-*5* (Fig. 5a), 3 for *dcl2*-*1* (Fig. 5b), 6 for *dcl3*-*1* (Fig. 5c) and 2 for *dcl4*-*2* (Fig. 5d). In case of *dcl1*-*5*, the majority (5) of these degradants displayed higher level of accumulation in mutant leaves than in wild type. Interestingly, in case of other analyzed mutants, all but one differentiating hcn-midi RNAs were less abundant than in wild type leaves. The only exception was one degradant more prevalent in *dcl3*-*1* than in wild type. Moreover, although only a limited number of changes reached the threshold of significance, it was evident that in *dcl2*-*1*, *dcl3*-*1* and *dcl4*-*2* the majority of hcn-midi RNAs accumulated to lower levels than in wild type.

**Fig. 6:**
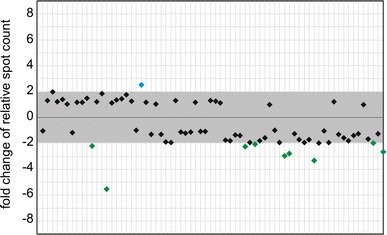
The relative levels of hcn-midi RNA accumulation in leaves of Arabidopsis exposed to salt stress and grown under standard conditions. The range of changes considered to be insignificant (change lower than twofold) is marked with a *gray box*. Each spot is represented by a separate *diamond symbol* (spots 1–70 are arranged consecutively from *left* to *right*). In order to determine the fold changes of hcn-midi RNA accumulation levels, the relative spot counts obtained for salinity-exposed leaves were divided by corresponding relative spot counts obtained for control leaves. Spots that displayed significantly higher relative intensity in control leaf profile are *colored green*, whereas a single spot that displayed significantly higher relative intensity in stressed leaf profile is *colored blue*

Until now, the accumulation of RNA degradants had mostly been studied in the context of stress-induced effects (Thompson et al. 2008; Haiser et al. 2008; Lee and Collins 2005; Li et al. 2008; Emara et al. 2010; Garcia-Silva et al. 2010). Therefore, we decided to test whether the accumulation pattern of hcn-midi RNAs may be influenced by abiotic stress. RNA samples, which were extracted from Arabidopsis rosette leaves and collected after a 6-h exposure to high salinity (150 mM NaCl), were subjected to standard 2D-PAGE analysis. The resultant autoradiograms were compared with those that were obtained earlier for unstressed rosette leaves (Fig. 6, Online Fig. 5). The undertaken analysis revealed 10 significantly differentiating degradants. Interestingly, almost all of stress-induced changes (9) were associated with a decrease in the level of hcn-midi RNA accumulation.

## Discussion

RNA degradation is a very effective and complex process that converts all types of transcripts into single ribonucleotides. However, growing evidence indicates that under specific conditions (e.g., stress or cancer transformation) certain types of RNA molecules are not completely digested (Thompson et al. 2008; Lee et al. 2009). As a result, stable intermediates of RNA degradation form and accumulate in cells. Cumulatively, these RNA degradants represent a set of molecules named the RNA degradome, which has not yet been well characterized (Jackowiak et al. 2011). Recently, it has been demonstrated that at least some of the RNA degradants can function as regulatory or signaling molecules (Haussecker et al. 2010; Lee et al. 2009; Zhang et al. 2009; Elbarbary et al. 2009; Yamasaki et al. 2009). These discoveries have raised a number of questions concerning the biogenesis and function of stable intermediates of RNA degradation. To learn more about these RNAs, we attempted to determine the effectiveness and specificity of their biogenesis under physiological conditions. The most fundamental problem that we were required to solve was how to distinguish degradants from other RNA molecules. Based on our earlier definition of the RNA degradome (Jackowiak et al. 2011), we decided to search for its members in the fraction of RNA that was longer than si/miRNAs but shorter than tRNAs (midi RNA). Within this range, we did not expect to find molecules that belonged to other classes of RNA. We found that numerous midi RNAs were repeatedly formed in plant leaf cells. The molecules that accumulated to the highest levels (i.e., comparable or higher than si/miRNAs) were easily visible on the 2D-gel autoradiograms as a characteristic pattern of spots. The analysis of midi RNAs that were classified as leaf-specific (2D-library) led to the identification of 67 RNA molecules. Except for a few intact tRNAs and unknown RNAs of intergenic origin, all of these RNAs were the products of the degradation of the following larger transcripts: tRNAs, rRNAs, mRNAs and snRNAs (Fig. 2). Interestingly, the composition of the 2D-library degradome did not correspond to the composition of total cellular RNA. For example, degradants that originated from rRNA constituted approximately 37 % of the 2D-library, whereas rRNA accounts for over 80 % of the total cellular RNA. A similar discordance between the compositions of the RNA degradome and the total RNA pool was also shown in other studies (Kawaji et al. 2008). This observation represents important evidence that the identified molecules were not the products of random RNA decay that occurred during sample handling. For the better assessment of midi RNAs diversity, we also analyzed the composition of the 1D-library. Because of the low number of molecules that have been sequenced thus far (750), statistical calculations are not conclusive. However, it is noteworthy that more than half of the hcn RNA degradants (from the 2D-library) were also present in the already-sequenced portion of the 1D-library. Although the 1D-library consisted of a more diversified population of midi RNAs, it was also dominated by tRNA and rRNA derivatives (Fig. 2, Online Table 2).

Based on the observations that have been made thus far by other researchers we could expect that some of the identified degradants may function as translational repressors. Recently, Ivanov et al. have shown that some tRNA degradants were capable of mediating translational inhibition in mammalian cells. The postulated mechanism assumed that 5′ tRNA fragments bound and displaced translation initiation factors (Ivanov et al. 2011). Among these degradants, 5′ fragments of tRNA^Ala^ and tRNA^Cys^ were particularly potent. Structure and function analyses revealed that this process was due to the presence of 5′-TOG motifs (i.e., four to five guanine residues) that are uniquely found at the 5′ ends of these tRNAs. In both libraries from Arabidopsis we identified several tRNA fragments that had 5′-TOG-like motifs. Out of them we selected tRNA degradants with 5, 4 or 3 guanine residues (tRNA^Ala^, tRNA^Leu^ and tRNA^Cys^, respectively) and tested their influence on in vitro translation. Taking into account the proposed mechanism of translational inhibition by tRNA fragments (Ivanov et al. 2011), we employed a plant in vitro translation system to facilitate the specific interaction between tRNA degradants and translation initiation factor(s). All selected degradants were able to block translation of reporter mRNA. A detailed elucidation of translational repression mechanism was certainly beyond the scope of our study. However, the obtained results suggest that the mechanism is similar to the one described earlier for human tRNA fragments (Ivanov et al. 2011). Interestingly, in contrast to published data (Ivanov et al. 2011), we did not observe a correlation between the number of guanine residues at 5′ end and inhibitory potential of a tRNA degradant. In addition, we demonstrated that human tRNA^Ala^, an effective translational repressor in rabbit reticulocyte lysate, was unable to block translation in wheat germ extract. This suggests that although some general mechanisms appear to be universal and conserved across kingdoms, there exist additional determinants that specifically regulate the potential of tRNA fragments to repress translation in animal and plant cells. Our results provide an important insight into the regulation of gene expression by tRNA degradants and encourage further analyses to better elucidate the phenomenon.

Our experiments prove that it is possible to routinely map hcn-midi RNAs using 2D-PAGE. Analyses of RNA samples that were isolated from Arabidopsis roots, leaves and flowers showed that some hcn-midi RNAs occurred in whole plants, while the formation of others was restricted to specific organs (see Fig. 4 and Online Fig. 3). As expected, the most distinctive pattern of hcn-midi RNA accumulation was observed in flowers, which are reproductive organs with high metabolic activity and profound changes in gene expression and RNA content.

Although stable intermediates of RNA degradation have been reported in many organisms (Thompson et al. 2008; Lee and Collins 2005; Jochl et al. 2008; Li et al. 2008; Zhang et al. 2009; Haiser et al. 2008; Garcia-Silva et al. 2010), their biological functions remain unclear. The potential connections of stable RNA degradants with cellular silencing networks have also been intensively investigated. A few independent studies have revealed that tRNA- and snoRNA-derived fragments display extensive similarity to small regulatory RNAs that are associated with the AGO and PIWI proteins (Ender et al. 2008; Taft et al. 2009; Kawamura et al. 2008; Lau et al. 2006; Haussecker et al. 2010; Couvillion et al. 2010; Ivanov et al. 2011) and exhibit a capacity for trans-silencing (Haussecker et al. 2010). It is also not clear if and how RNA degradants depend on components of the RNAi machinery, and in most cases, the mechanism for their biogenesis is questioned. The biogenesis of tRNA derivatives has been the most studied, and two ribonucleases have been recently shown to participate in the production of tRNA halves after oxidative stress stimuli: Rny1 in *Saccharomyces* *cerevisiae* (Thompson and Parker 2009) and Angiogenin in human cells (Yamasaki et al. 2009). Other studies have also shown that Dicer is required for the biogenesis of a small RNAs that originate from tRNA and snoRNAs in human cells (Ender et al. 2008; Cole et al. 2009), fruit flies and mice (Taft et al. 2009; Babiarz et al. 2008).

To determine if dysfunction of Dicer-like proteins influenced the accumulation of hcn-midi RNAs in plants we compared 2D-gel images of the hcn-midi RNAs from wild type and four *dcl* mutant plants (Fig. 5 and Online Fig. 4). Interestingly, this analysis revealed only mild differences between the plants (smaller than between different organs of a wild type plant). This may result from partially redundant functions of Dicer-like proteins in Arabidopsis (Gasciolli et al. 2005). Nevertheless, in all mutants with the exception of *dcl1*-*5*, a tendency towards a reduction of hcn-midi RNA accumulation levels was observed. DCL1 ribonuclease is mainly involved in the processing of pre-miRNAs into mature miRNAs (Kurihara and Watanabe 2004). Our data suggest that the impairment of miRNA biogenesis does not significantly hamper the generation of hcn-midi RNAs. Out of 6 degradants whose accumulation levels were significantly changed (in comparison to wild type) 5 occurred in higher concentration in *dcl1*-*5* mutant. These changes could result from the abolishment of DCL-1-dependent processing of some RNA species (e.g., tRNA or rRNA). In case of *dcl3*-*1*, *dcl2*-*1* and *dcl4*-*2* only several changes reached the significance threshold and were associated with a reduced prevalence of hcn-midi RNAs in comparison to wild type (with one exception in *dcl3*-*1*). Despite evidence showing that Dicer can be involved in the cleavage of tRNAs and snoRNAs (Taft et al. 2009; Ender et al. 2008; Cole et al. 2009; Babiarz et al. 2008), our results suggest that dysfunction of DCL ribonucleases does not considerably affect the production of hcn-midi RNAs in Arabidopsis. Based on this observation one can hypothesize that the pathways of small regulatory RNA and hcn-midi RNA generation in Arabidopsis are generally unrelated. Apparently, enzymes other than DCLs are responsible for the continuous production of these RNAs.

As mentioned above, the formation of stable RNA degradation intermediates commonly implies a stress-induced event. Our 2D-analyses of midi RNA fractions, which were obtained from the leaves of salinity-exposed Arabidopsis, also revealed stress-induced changes in the patterns of accumulation of hcn-midi RNAs. Interestingly, all significant changes were associated with a decrease in the level of hcn-midi RNA accumulation in comparison with plants grown under standard conditions.

The observations presented in this paper show that plant cells contain a complex population of stable RNA degradation intermediates. These molecules are generated not only as a result of stress as it has earlier been postulated but also under physiological conditions. The data collected suggest that the pattern of hcn RNA degradant accumulation is organ-specific. In addition, our translation inhibition experiments reinforce the idea that RNA degradants may possess important regulatory functions. However, it remains to be experimentally demonstrated which elements of cellular pathways are regulated by RNA degradants and which RNAs and/or proteins are also involved in these processes.

## Electronic supplementary material

Below is the link to the electronic supplementary material.

Supplementary material 1 (XLSX 37 kb)

Supplementary material 2 (XLSX 77 kb)

Supplementary material 3 (PDF 4080 kb)
