# Supplementary material for: Identification of stable, high copy number, medium-sized RNA degradation intermediates that accumulate in plants under non-stress conditions
Source: Plant Mol Biol. 2013 May 25;83(3):191–204. doi: 10.1007/s11103-013-0079-3 (PMC3777163; doi:10.1007/s11103-013-0079-3)
Supplement: Supplementary file 3 — Supplementary material 3 (PDF 4080 kb) [file 11103_2013_79_MOESM3_ESM.pdf]

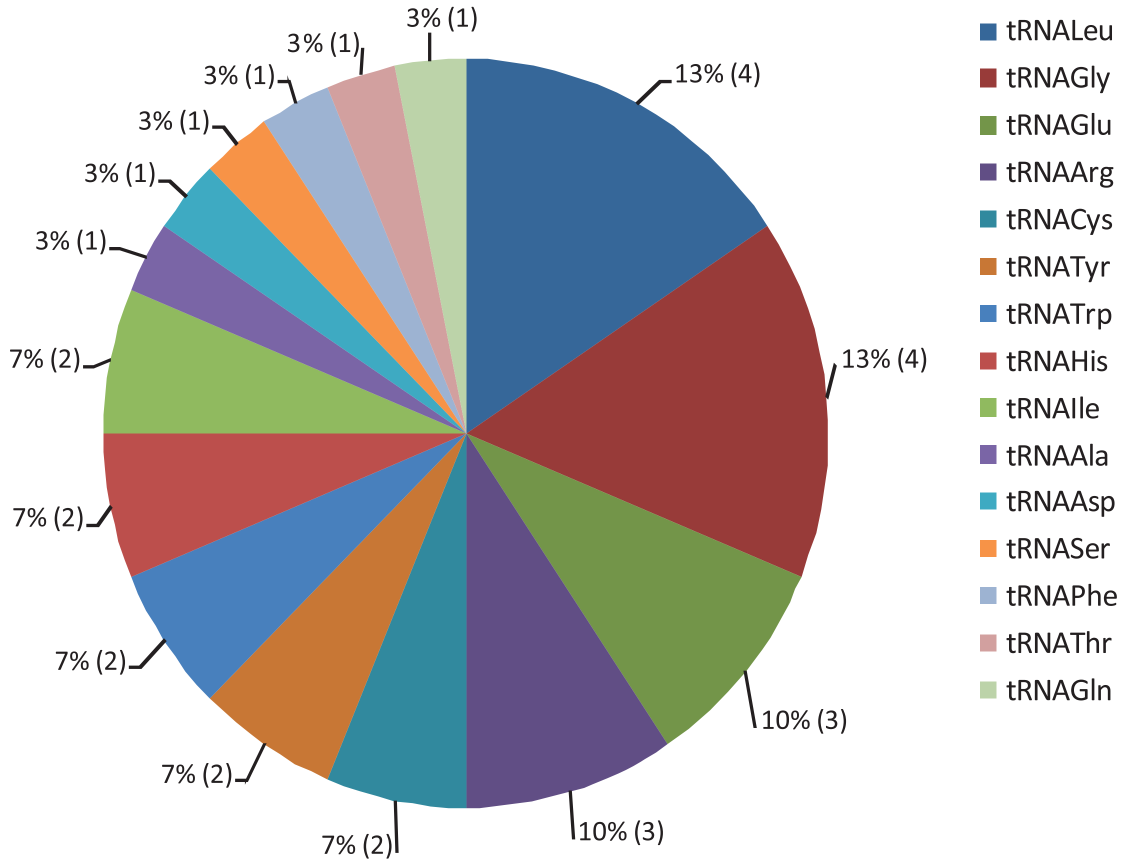

Online Fig. 1 Contribution of particular tRNA-derived fragments to the tRNA degradome in the 2D-library. Number of identified molecules of each tRNA presented in parentheses

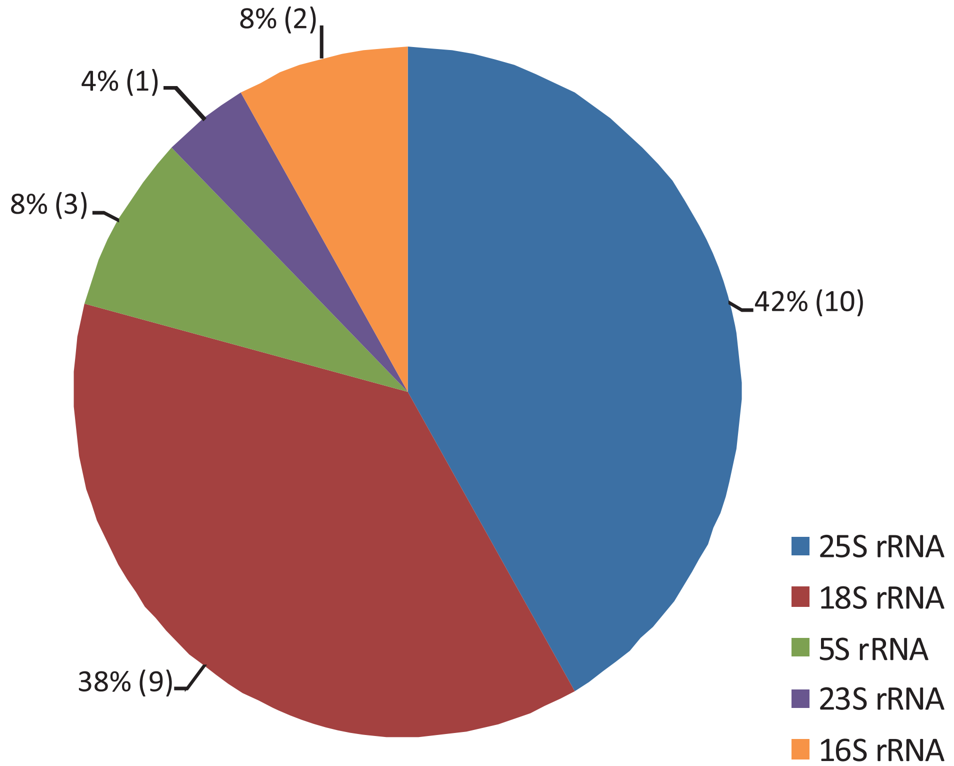

Online Fig. 2 Contribution of particular rRNA-derived fragments to the rRNA degradome in the 2D-library. Number of identified molecules of each rRNA is presented in parentheses

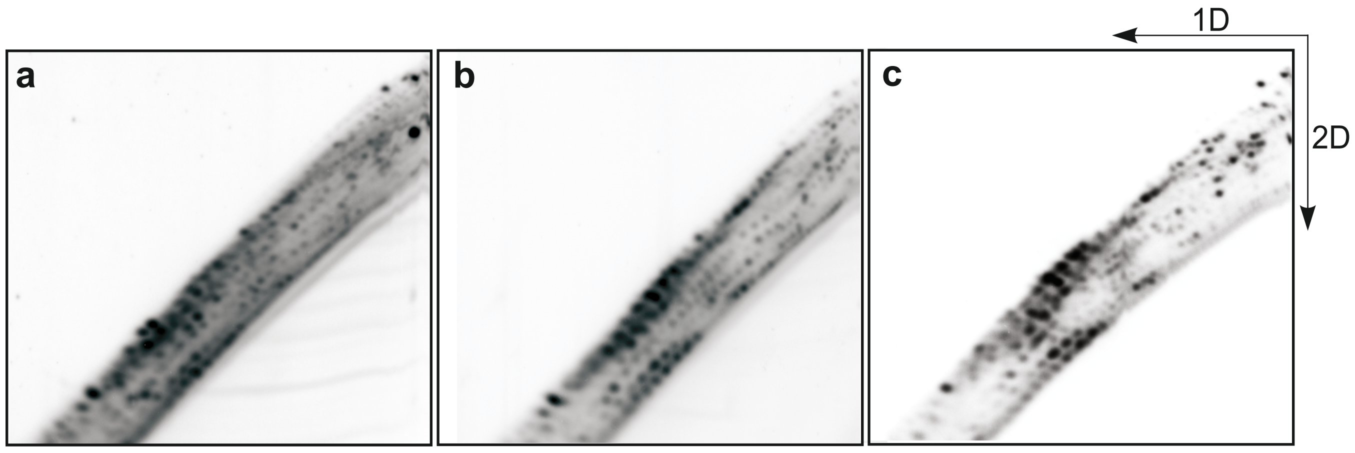

Online Fig 3 Comparative 2D-PAGE analysis of the midi RNA fraction isolated from Arabidopsis rosette leaves (a), roots (b) and flowers (c). The directions of electrophoresis in the first and second dimension are indicated by arrows

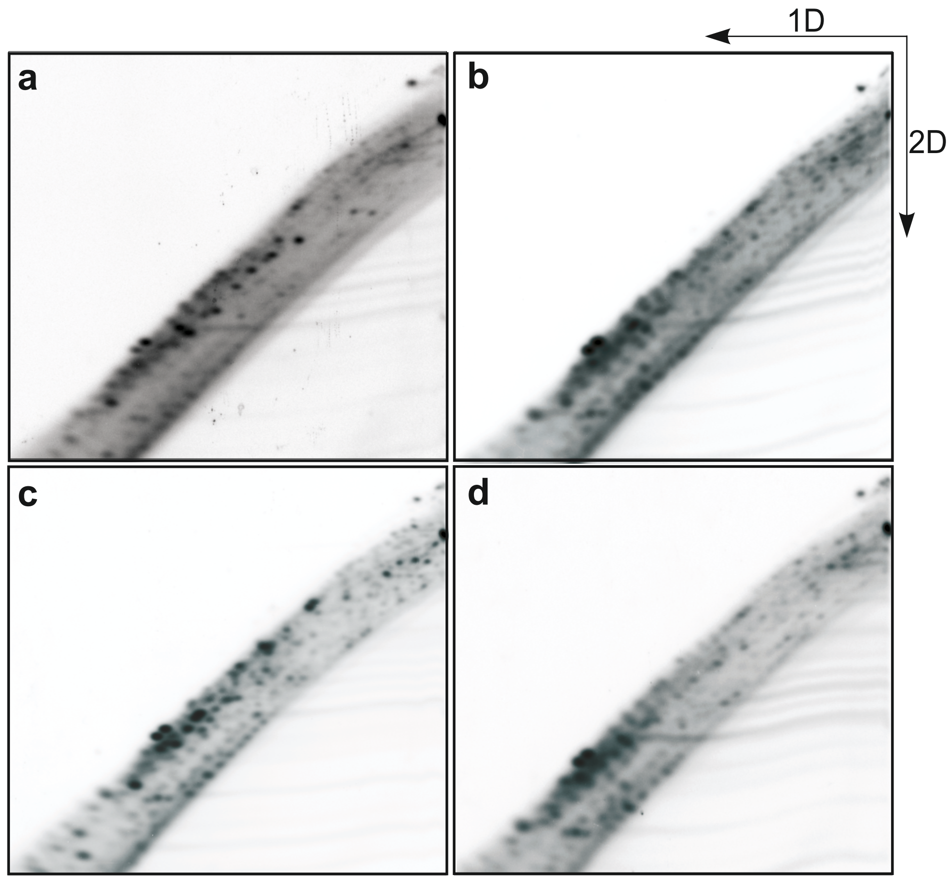

Online Fig 4 Comparative 2D-PAGE analysis of the midi RNA fraction isolated from Arabidopsis *dcl* mutants: *dcl1-5* (a), *dcl2-1* (b), *dcl3-1* (c), and *dcl4-2* (d). The directions of electrophoresis in the first and second dimension are indicated by arrows

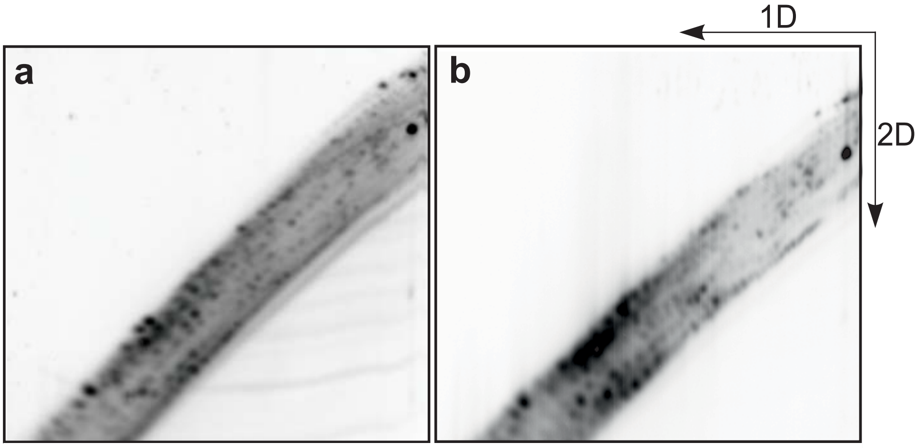

Online Fig 5 Comparative 2D-PAGE analysis of the midi RNA fraction isolated from leaves of Arabidopsis grown under standard condition (a) and exposed to salinity (b). The directions of electrophoresis in the first and second dimension are indicated by arrows
